# Halitosis and Quality of Life in Young Orthodontic Patients: A Cross-Sectional Assessment of Mouthwash Use and Traditional, Rotative, and Sonic Toothbrushes

**DOI:** 10.3390/medicina61050815

**Published:** 2025-04-28

**Authors:** Hamsah Musa, Ioana Georgiana Pașca, Malina Popa, Octavia Bălean, Ramona Dumitrescu, Ruxadra Sava Roșianu, Atena Gălușcan, Roxana Oancea

**Affiliations:** 1 Doctoral School, Faculty of Dental Medicine, “Victor Babes” University of Medicine and Pharmacy Timisoara, Revolutiei Boulevard 9, 300041 Timisoara, Romania; musa.hamsah@umft.ro; 2 Department of Pediatric Dentistry, Faculty of Dental Medicine, “Victor Babes” University of Medicine and Pharmacy Timisoara, Revolutiei Boulevard 9, 300041 Timisoara, Romania; 3 Translational and Experimental Clinical Research Centre in Oral Health, Department of Preventive, Community Dentistry and Oral Health, Faculty of Dental Medicine, “Victor Babes” University of Medicine and Pharmacy Timisoara, Revolutiei Boulevard 9, 300041 Timisoara, Romania; balean.octavia@umft.ro (O.B.); dumitrescu.ramona@umft.ro (R.D.); sava-rosianu.ruxandra@umft.ro (R.S.R.); galuscan.atena@umft.ro (A.G.); roancea@umft.ro (R.O.)

**Keywords:** halitosis, orthodontics, oral hygiene, clinical study, chlorhexidine, toothbrush, mouthwash, caries risk, quality of life

## Abstract

*Background and Objectives*: Halitosis is common among orthodontic patients, potentially exacerbated by plaque retention around brackets. It was hypothesized that patients using sonic toothbrushes would report lower halitosis impact scores compared to those using traditional or rotative toothbrushes. This study aimed to compare the Halitosis-Associated Life Quality Test (HALT) and Short Form (SF-36) domains among different toothbrush users and to evaluate mouthwash subgroups and “during” vs. “after” appliance removal. *Methods*: Based on a power calculation (effect size f = 0.30, α = 0.05, 1 − β = 0.80), 174 patients were required. A total of 174 orthodontic patients (57 traditional, 64 rotative, and 53 sonic; mean age 18.0 ± 1.5 years) completed the Halitosis-Associated Life Quality Test (HALT), SF-36, and organoleptic assessments. *Results*: Sonic toothbrush users reported significantly lower HALT scores (34.8 ± 5.8) vs. rotative (38.1 ± 6.0) and traditional (42.7 ± 6.2) toothbrush users. Spearman’s correlation (r = −0.49 to +0.54) demonstrated that sonic brushing had a moderate negative relationship with halitosis scores, indicating lower malodor for this technique. Patients using chlorhexidine-based mouthwash had the most favorable HALT (34.3 ± 5.7) and organoleptic (1.5 ± 0.4) scores. Those who completed orthodontic treatment showed better outcomes than active treatment patients. *Conclusions*: Optimized plaque control with sonic brushing and chlorhexidine-based mouthwash correlates with reduced halitosis and improved quality of life during orthodontic treatment.

## 1. Introduction

Halitosis, or bad breath, arises from multiple etiologies, often related to the production of volatile sulfur compounds (VSCs) by oral bacteria [1]. In orthodontic patients, fixed appliances can complicate daily plaque control, creating niches for bacterial overgrowth [2]. Consequently, halitosis may increase, adversely affecting psychosocial confidence and overall quality of life [3]. Several investigations highlight the need for improved oral hygiene protocols in orthodontic populations due to elevated plaque levels [4,5].

Toothbrushing remains the fundamental strategy for plaque removal, but the brush design and technology can significantly influence outcomes [6,7]. Traditional manual toothbrushes depend on patient technique and motivation, whereas electric brushes—rotative and sonic—deliver more consistent movements, potentially offering superior plaque disruption [8]. A growing body of evidence suggests that advanced mechanical plaque control devices, including rotative and sonic toothbrushes, offer enhanced plaque removal and potentially reduce halitosis in various dental contexts, including orthodontics [9]. However, direct comparisons among these brushes remain limited.

Alongside mechanical cleaning, mouthwashes are commonly recommended to reduce microbial load [10]. Chlorhexidine-based solutions, for instance, have robust antibacterial properties, showing efficacy against the oral pathogens responsible for malodor [11]. Essential oil-based rinses present a more natural alternative and have demonstrated moderate efficacy for controlling plaque and halitosis [12]. Fluoride-based mouthwashes offer caries-preventive benefits but may be less targeted toward halitosis, although they can still bolster overall oral health [13].

Fixed orthodontic appliances can remain in place for 1–3 years, during which the risk of plaque accumulation is significantly elevated [14]. Moreover, inadequate plaque control during orthodontic therapy elevates caries risk, as biofilm accumulation can lead to demineralization around brackets and wires [15]. This heightened risk underscores the importance of both effective brushing techniques and adjunctive mouthwash use to maintain gingival health, prevent caries, and minimize malodor. Therefore, clinical success hinges on patient education and adherence to recommended devices and adjuncts, including suitable toothbrush types and mouthwashes [16].

Quality-of-life measures, such as the Halitosis-Associated Life Quality Test (HALT) and the SF-36, provide insight into both the specific impact of malodor and overall physical-mental health [17,18,19]. Their combined application in orthodontic research can unveil how halitosis influences social interactions, emotional well-being, and functional status [19]. Moreover, the “during” versus “after” appliance phase comparison illuminates whether successful plaque management persists once brackets are removed [20].

We hypothesized that sonic toothbrush users would demonstrate significantly lower HALT and organoleptic scores than patients using traditional or rotative brushes. We further hypothesized that mouthwash use—especially chlorhexidine-based mouthwash—would correlate with decreased halitosis and enhanced quality of life. In this cross-sectional study, we aimed to (i) compare HALT and SF-36 scores among orthodontic patients using traditional, rotative, and sonic toothbrushes; (ii) evaluate differences based on mouthwash use and type; and (iii) investigate disparities in halitosis and quality of life between those actively undergoing treatment versus those in retention or post-treatment phases. Our overarching goal is to guide clinicians toward optimal oral hygiene protocols that mitigate halitosis and maximize patient well-being.

## 2. Materials and Methods

### 2.1. Study Design and Ethical Considerations

This non-randomized cross-sectional study was undertaken at multiple orthodontic clinics, including those affiliated with the “Victor Babeș” University of Medicine and Pharmacy, Timișoara, with consecutive enrollment of subjects who met the inclusion criteria. No randomization was conducted because we aimed to observe real-world usage patterns of different toothbrushes. The recruitment period ranged from January 2023 to January 2025. We adhered to the Declaration of Helsinki guidelines, ensuring informed consent from all participants. Written informed consent was obtained from all participants; for minors, additional consent was provided by a parent or legal guardian.

Electronic medical records were examined to retrieve demographic and clinical information, while direct evaluations were conducted to gather detailed functional and quality-of-life data. Patient confidentiality was maintained through secure data management protocols in strict accordance with international regulations, including the EU GCP. All participants provided written informed consent, as mandated by national legal requirements (Article 167 of Law No. 95/2006 and Order 904/2006, Article 28, Chapter VIII).

The inclusion criteria included: (1) age 14–28 years, (2) under or recently completed fixed orthodontic treatment, (3) no significant systemic conditions affecting oral health, and (4) willingness to complete questionnaires and clinical assessments. The exclusion criteria were considered as the following: (1) antibiotic use within the previous 4 weeks, (2) severe periodontal disease requiring specialist treatment, (3) known allergies to mouthwash ingredients, and (4) smoking ≥5 cigarettes/day. These criteria ensured a representative orthodontic sample while controlling for major confounders like heavy smoking or severe periodontal involvement.

### 2.2. Participants and Data Collection

Prior to data collection, we performed a power analysis (G*Power 3.1 software). We assumed a medium effect size (**f** = 0.30) based on pilot data indicating clinically meaningful differences in HALT scores among toothbrush groups. At an alpha level of 0.05 and desired power of 0.80, we required a minimum of 159 participants. Allowing for potential dropouts, we set our final sample target to 174.

A total of 174 orthodontic patients were recruited: 57 consistently used traditional manual toothbrushes, 64 used rotative electric brushes, and 53 used sonic brushes. Each participant provided demographic data (age and sex) and relevant dental history. The status of orthodontic treatment (“during” vs. “after” appliance removal) was recorded alongside treatment duration in months. All individuals completed the Halitosis-Associated Life Quality Test (HALT), an organoleptic assessment, and the SF-36 survey. Data on mouthwash use (yes/no) and mouthwash type (chlorhexidine-based, essential oil-based, and fluoride-based) were documented. To minimize variability, participants were instructed to abstain from eating, drinking, or using any oral hygiene product for at least two hours before examination.

### 2.3. Toothbrushes and Mouthwashes

Details of the toothbrushing devices used in the study included three types, each supplied or recommended directly by the clinic to ensure uniformity in brand and features among participants within each group. The traditional brush was a soft-bristled, flat-trim manual brush with a small head. The rotative brush referred to a commercially available oscillating-rotating brush, specifically the Oral-B Professional, which features a round head and approximately 7600 oscillations per minute. The sonic brush was a popular model, the Philips Sonicare, known for delivering about 31,000 strokes per minute.

The mouthwash protocol involved three different types of mouthwashes. The first was a chlorhexidine-based mouthwash containing 0.12% CHX, with a recommended rinse duration of 30 s, to be used twice daily. The second was an essential oil-based mouthwash, specifically Listerine, with a recommended usage of 20 mL for 30 s, also twice daily. The third type was a fluoride-based mouthwash containing 0.05% sodium fluoride, brand Y, recommended at 20 mL for 30 s, but only once daily at bedtime. Participants were instructed on the proper rinsing technique and timing, such as avoiding immediate rinsing with water after use, and compliance was monitored based on self-reports during monthly visits.

### 2.4. Questionnaires and Organoleptic Assessment

The HALT comprises 20 items scored on a 0–5 scale, yielding a total range of 0–100, where higher scores indicate a stronger negative impact of halitosis on daily life. The SF-36 questionnaire evaluates eight domains of general health, each ranging from 0 to 100 (higher indicates better self-reported health). Both have established psychometric reliability and validity in oral health research [17,18]. The Romanian version of these questionnaires was administered to study participants.

We employed a single calibrated examiner for all organoleptic evaluations to minimize inter-rater variability. Calibration was performed through a 2 h training session using standardized breath samples (0–5 scale) under the guidelines recommended by Rosenberg [1], focusing on consistency in rating. Prior to the main study, intra-rater reliability was assessed on 15 pilot participants, yielding a Cohen’s kappa of 0.86, indicating excellent agreement.

All participants received standardized training on their assigned oral hygiene protocol. This included a 5 min demonstration of correct brushing technique (traditional, rotative, or sonic) by a dental hygienist. For electric brushes (rotative or sonic), we provided instructions on brush head positioning, recommended duration (2 min), and the manufacturer’s guidelines for pressure and motion. Participants were asked to return for monthly check-ups, during which adherence (self-reported frequency and technique) was discussed and reinforced. Adherence to the recommended oral hygiene protocol was further checked using a simple questionnaire during each visit (e.g., “How many times per day did you brush?”; “Did you follow the 2-min rule?”).

Each participant was instructed to refrain from eating, drinking (other than water), or using any oral hygiene product for at least 2 h before the exam. During the assessment, the examiner stood approximately 10 cm from the participant’s mouth after participants closed their mouths and breathed through their nose for three minutes. The 0–5 scoring criteria were adapted from established clinical guidelines: Score 0: No odor; Score 1: Barely noticeable odor; Score 2: Slight but clearly noticeable odor; Score 3: Moderate malodor; Score 4: Strong malodor; Score 5: Extremely strong malodor, detectable instantly.

To further reduce examiner bias, the examiner wore a nose clip between assessments of different participants to recover from any lingering olfactory stimuli (a “reset” period of 2 min). We also standardized the testing environment (well-ventilated, odor-free room) and used unscented personal protective equipment to avoid external odors.

### 2.5. Statistical Analysis

Data were analyzed using SPSS v28 (IBM Corp., Armonk, NY, USA). Figure generation was carried out using GraphPad Prism 9 (GraphPad Software, San Diego, CA, USA). We applied default settings for axis scaling and color schemes, ensuring consistency across charts and minimizing the risk of misrepresenting data. Continuous variables underwent normality testing (Shapiro–Wilk). One-way ANOVA with Tukey’s post hoc test was performed for comparing HALT and SF-36 scores among toothbrush types (traditional, rotative, sonic). Independent t-tests and one-way ANOVA were used for subgroup analyses (mouthwash use, mouthwash type, and “during” vs. “after” appliances). All data are expressed as mean ± SD with one decimal place. A Spearman’s correlation matrix examined relationships among HALT scores, SF-36 Social Functioning scores, organoleptic scores, age, and treatment duration (*p* < 0.05 considered significant). Effect sizes were interpreted as small (r < 0.30), moderate (0.30–0.59), or strong (≥0.60). *p*-values were reported for all comparisons to ensure transparency in statistical significance.

## 3. Results

### Patient Demographics

Table 1 displays the baseline demographics and orthodontic status of our 174 participants by their preferred toothbrush type. The mean age across all groups was approximately 18 years (*p* = 0.377). Gender distribution did not differ significantly by toothbrush choice, with slightly more females overall (61.5%). Treatment duration was also comparable, averaging around 14.8 months (*p* = 0.460). Regarding orthodontic stage, 56.3% remained in active treatment (“during”), while 43.7% had completed therapy within six months prior to the study (“after”). The proportions of active vs. post-treatment patients did not significantly differ among the three groups (*p* = 0.679).

Table 2 presents the mean HALT scores across four distinct domains—Emotional Impact, Social/Interactional, Personal/Functional, and Physical Concerns—as well as the total HALT scores. Statistical differences among the three toothbrush groups were evident in all domains. Traditional brush users consistently registered the highest scores. By contrast, sonic brush users reported the lowest scores, suggesting fewer halitosis-related concerns. Specifically, the total HALT scores were 42.7 ± 6.2 for the traditional group, 38.1 ± 6.0 for the rotative group, and 34.8 ± 5.8 for the sonic group (*p* < 0.001). Post hoc comparisons showed that each group significantly differed from the others, with the largest difference noted between traditional and sonic users (*p* < 0.001).

Table 3 presents the SF-36 domain scores across three different toothbrush groups: traditional, rotative, and sonic. The sample sizes for each group were 57, 64, and 53, respectively. The domains measured included Physical Functioning, Role Physical, Bodily Pain, General Health, Vitality, Social Functioning, Role Emotional, and Mental Health. For Physical Functioning, the scores were 82.3 ± 5.3 for the traditional group, 83.9 ± 4.4 for the rotative group, and 85.1 ± 5.2 for the sonic group, with a *p*-value of 0.046. The Role Physical scores were 78.5 ± 7.1, 80.3 ± 6.2, and 81.9 ± 6.4 for traditional, rotative, and sonic groups, respectively, with a *p*-value of 0.049.

For Bodily Pain, scores were reported as 76.7 ± 6.8 for traditional, 78.0 ± 6.4 for rotative, and 79.8 ± 6.2 for sonic, with a *p*-value of 0.036. In General Health, scores of 73.8 ± 6.2, 76.6 ± 5.7, and 77.7 ± 6.0 were noted for the traditional, rotative, and sonic groups, respectively, resulting in a *p*-value of 0.031. The Vitality scores were 75.3 ± 5.6, 77.6 ± 5.1, and 79.0 ± 5.0 for the respective groups, with a *p*-value of 0.01. The scores for Social Functioning were 80.0 ± 4.5 for traditional, 82.2 ± 4.3 for rotative, and 84.3 ± 4.2 for sonic, with a significant *p*-value of 0.001. The Role Emotional and Mental Health domains also showed differences, with scores of 76.8 ± 6.2, 77.7 ± 5.9, and 79.3 ± 5.6 for Role Emotional and 79.3 ± 5.7, 80.5 ± 5.4, and 82.1 ± 5.2 for Mental Health, with *p*-values of 0.098 and 0.046, respectively.

Table 4 highlights the key outcome differences between patients who regularly used mouthwash (*n* = 102) and those who did not (*n* = 72). A significantly lower mean HALT total score (35.1 ± 6.0 vs. 39.2 ± 6.3, *p* < 0.001) among mouthwash users indicated a reduced subjective burden of halitosis. This aligned with their lower mean organoleptic score (1.7 ± 0.4 vs. 2.1 ± 0.5, *p* < 0.001), suggesting that mouthwash usage effectively suppresses malodor. Additionally, mouthwash users exhibited higher SF-36 Social Functioning scores (83.7 ± 4.3 vs. 80.2 ± 4.8, *p* = 0.002). This difference underscores the potential psychosocial benefits of improved oral hygiene—namely, heightened confidence in social environments.

Table 5 compares halitosis and quality-of-life measures among orthodontic patients using three different mouthwash formulations. The ANOVA indicated a statistically significant variation in both HALT total scores (*p* = 0.039) and organoleptic ratings (*p* = 0.033). Chlorhexidine-based mouthwash users reported the lowest mean HALT score (34.3 ± 5.7) and organoleptic score (1.5 ± 0.4), supporting existing evidence that chlorhexidine effectively targets oral pathogens linked to malodor. Essential oil-based mouthwash yielded intermediate improvements (35.7 ± 5.9 HALT; 1.8 ± 0.5 organoleptic), while the fluoride-based rinse produced slightly higher HALT (36.4 ± 5.6) and organoleptic (1.9 ± 0.4) scores. Although essential oil-based and fluoride-based mouthwashes were not significantly different from each other (*p* = 0.515), chlorhexidine vs. fluoride-based reached significance (*p* = 0.046) in the Tukey post hoc analysis.

**Figure 1:**
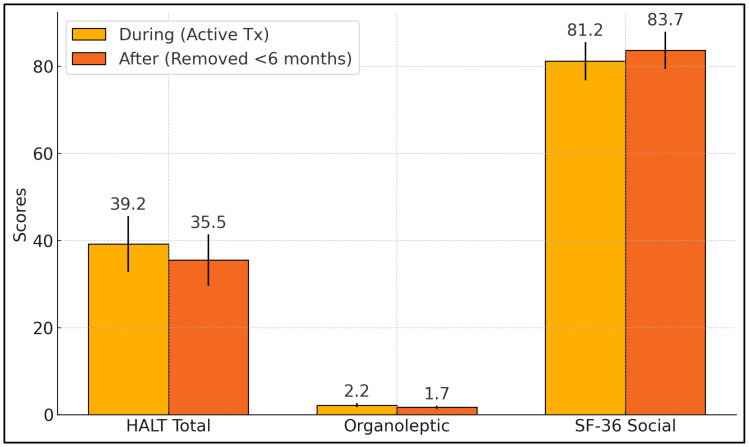
Comparison of “during” vs. “after” orthodontic appliance removal.

Table 6 compares patients still wearing orthodontic appliances (“during”, *n* = 98) to those who completed treatment within the past six months (“after”, *n* = 76). Statistically significant differences emerged in HALT total scores (*p* = 0.002), organoleptic ratings (*p* < 0.001), and SF-36 Social Functioning scores (*p* = 0.015). On average, active treatment patients reported higher HALT scores (39.2 ± 6.4) than those in the post-treatment phase (35.5 ± 5.9), indicating a more substantial halitosis burden among patients with fixed brackets. Likewise, organoleptic scores were higher in the active group (2.2 ± 0.5 vs. 1.7 ± 0.4), aligning with the rationale that orthodontic hardware can promote plaque retention and consequent malodor. SF-36 Social Functioning scores were notably lower in the active treatment group (81.2 ± 4.4) compared to their counterparts (83.7 ± 4.3, *p* = 0.015), underscoring how halitosis may hinder social interactions during active orthodontic therapy (Figure 1).

**Figure 2:**
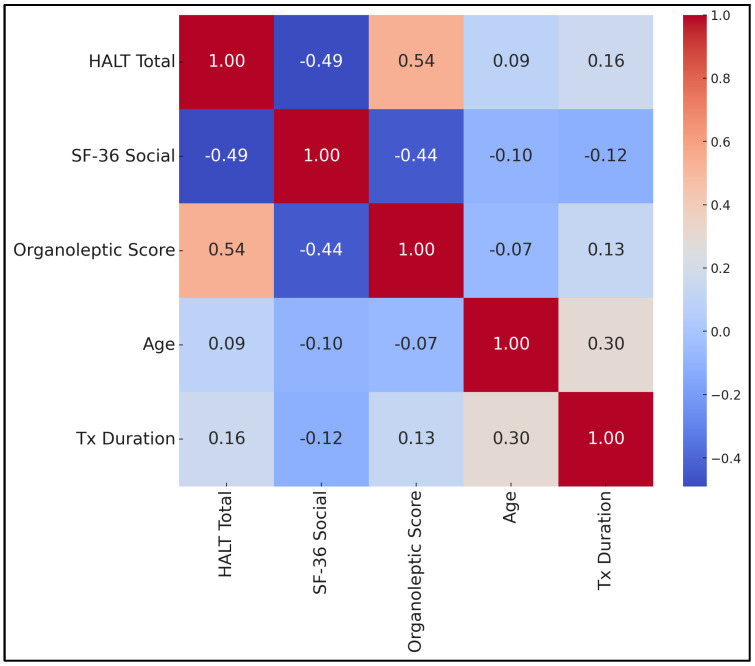
Correlation matrix.

Table 7 and Figure 2 present a comprehensive correlation matrix among HALT total score, SF-36 Social Functioning score, organoleptic score, age, and treatment duration. Notably, HALT total score correlated moderately and positively with organoleptic score (r = +0.54, *p* < 0.001), suggesting that increases in clinically assessed malodor coincide with higher self-reported halitosis burden. By contrast, HALT total score showed a moderately negative correlation with SF-36 Social Functioning score (r = −0.49, *p* < 0.001), indicating that higher halitosis complaints aligned with poorer perceived social well-being.

Organoleptic score also correlated negatively with SF-36 Social Functioning score (r = −0.44, *p* < 0.001), emphasizing how objectively measured bad breath can diminish social interactions. Age exhibited a small positive correlation with treatment duration (r = +0.30, *p* < 0.001), reflecting that older participants in this sample tended to have slightly longer orthodontic timelines but did not strongly link with halitosis metrics. The relatively small correlations for HALT score vs. age (r = +0.09) and organoleptic score vs. age (r = −0.07) implied minimal age-related differences in perceived or measured malodor.

Moreover, a moderately negative correlation existed between sonic brushing (coded as 1 = yes, 0 = no) and HALT score (r = −0.42, *p* < 0.001), indicating that the more participants relied on sonic brushes, the lower their reported halitosis burden. By contrast, a positive correlation was found between traditional brushing (coded 1 = yes, 0 = no) and HALT score (r = +0.38, *p* = 0.001), consistent with higher malodor complaints.

## 4. Discussion

The current study confirmed the initial hypothesis that sonic toothbrushes correlate with lower HALT and organoleptic scores compared to both rotative and traditional brushes. Although no randomization was used due to the cross-sectional nature of the study, we accounted for potential confounders (e.g., mouthwash use and treatment phase) through subgroup analyses.

Surprisingly, some participants in the post-treatment group still exhibited moderate to high halitosis scores, with organoleptic scores of 2 or higher. Several factors may have contributed to this outcome. Firstly, the residual effects of prolonged treatment could be a factor, where extended bracket wear might lead to gingival inflammation or areas of plaque stagnation that persist even after the removal of the appliance. Secondly, interviews revealed that 30% of the participants in the “after” group (*n* = 23) did not adhere to the prescribed oral hygiene regimen, leading to inconsistent brushing and mouthwash use. Lastly, about 12% of the post-treatment patients (*n* = 9) reported experiencing mild xerostomia during the retention phase, which could be attributed to altered salivary flow in post-orthodontic therapy. This suggests that further investigations are warranted to establish a direct link between decreased salivary flow and persistent malodor. When examining all subgroups, the lowest mean HALT (34.3 ± 5.7) and organoleptic (1.5 ± 0.4) scores were found among sonic + chlorhexidine users who had also completed treatment, suggesting these variables collectively shape malodor outcomes. Clinically, this aligns with existing literature advocating for comprehensive hygiene strategies (proper brushing, effective rinsing, and follow-up compliance) to minimize halitosis throughout and beyond orthodontic therapy.

The subgroup analyses underscored the importance of mouthwash usage, revealing significantly lower HALT and organoleptic scores among users than non-users. The chlorhexidine-based rinse proved to be most effective, consistent with the literature showing its potent antibacterial action and halitosis reduction [10,11]. While essential oil-based and fluoride-based mouthwashes also offered improvements, the differences were more modest. Additionally, patients who had completed orthodontic treatment exhibited reduced malodor and better social well-being than those still in active therapy. This outcome aligns with evidence that plaque retention and resultant halitosis often diminish once brackets are removed, although good oral hygiene practices remain necessary to sustain improvements [4,14].

The correlation matrix confirmed moderate interrelationships among HALT, SF-36 Social Functioning, and organoleptic scores, suggesting that halitosis impinges notably on social and emotional domains. Notably, age and treatment duration correlated modestly (r = +0.30), indicating that older participants generally had lengthier treatments, yet age did not significantly predict halitosis severity. Despite the robust cross-sectional data, caution is warranted regarding causal inferences. Future longitudinal or randomized controlled studies might track halitosis outcomes from orthodontic initiation through post-removal, comparing different brushing and mouthwash regimens. Moreover, employing objective gas chromatography could strengthen measurement accuracy, complementing our organoleptic findings. Overall, our data underscore that improving mechanical plaque control and encouraging targeted mouthwash use can significantly mitigate halitosis, thereby enhancing key domains of patient-reported quality of life.

In the field of orthodontic care, maintaining optimal oral hygiene is a critical challenge due to the complexity introduced by fixed appliances, which trap food particles and obstruct mechanical brushing. Two studies by Christina Erbe et al. [21,22] delved into this issue by evaluating the efficacy of different toothbrushes in plaque removal among orthodontic patients. The first study conducted a randomized clinical trial to compare an oscillating-rotating electric toothbrush with a sonic toothbrush using digital imaging analysis. It was found that both toothbrushes effectively reduced plaque, which initially covered more than 50% of the tooth area, but the oscillating-rotating toothbrush achieved a statistically significant greater reduction in plaque (*p* = 0.017) compared to the sonic toothbrush. Similarly, a subsequent study expanded on these findings by comparing three different brushing modalities: an oscillating-rotating toothbrush with a specialized orthodontic brush head, the same handle with a regular brush head, and a standard manual toothbrush. The results demonstrated that the electric toothbrush, particularly with the orthodontic brush head, removed significantly more plaque than the manual toothbrush, with mean differences of 6% [4.4–7.6%] for the orthodontic head and 3.8% [2.2–5.3%] for the regular head, underscoring the superiority of specialized electric brush heads in managing oral hygiene for orthodontic patients.

In correlation to our findings, in the realm of oral health research, recent studies have elucidated the significant impact of halitosis on oral-health-related quality of life (OHRQoL), as well as explored interventions to manage this condition among specific populations. A systematic review and meta-analysis by Luisa Schertel Cassiano et al. [23] demonstrated a clear association between halitosis and impaired OHRQoL across a pooled sample of 2692 individuals from ten cross-sectional studies (SMD 0.51; 95% confidence interval 0.27–0.75). This meta-analysis highlighted that halitosis significantly affects adults’ quality of life, irrespective of the assessment method or cultural background, although methodological quality did contribute to some study heterogeneity, explaining 20% of the variance observed. In a similar manner, the study by Konstantina Tsironi et al. [24] found that mastic mouthwash significantly reduced halitosis, measured by hydrogen sulfide levels (from 221.00 ppb to 125.00 ppb), in adolescents undergoing orthodontic treatment, indicating a potential therapeutic benefit (coef: 72.34, 95% CI: 8.48, 136.27, *p* = 0.03). However, no significant changes were noted in other volatile sulfur compounds or in subjective measures of malodor. These studies collectively underscore the psychological and social dimensions of halitosis, suggesting that while treatment can reduce the biochemical markers of halitosis, its perceptual aspects might require different or additional interventions.

In recent research exploring oral health management in orthodontic patients, two studies offer insights into the effectiveness of different hygiene tools. A randomized clinical trial by Ioulia-Maria Mylonopoulou et al. [25] compared the efficacy of electric three-dimensional (3D) toothbrushes against manual toothbrushes in plaque removal and reducing gingival inflammation in adolescents with fixed orthodontic appliances. Despite rigorous testing over three months, the study concluded that there was no significant difference between the two types of toothbrushes in improving oral hygiene (*p* = 0.89 for Modified Silness and Löe plaque index interaction), suggesting that orthodontists should instead focus on promoting better dental care practices regardless of the brush type used. In a similar manner, the systematic review by Matheus Melo Pithon et al. [26] assessed the effectiveness of various oral mouthwashes in reducing cariogenic biofilm in orthodontic patients. This review, encompassing 15 studies, concluded that oral antiseptics, including chlorhexidine, cetylpyridinium, and several other formulations, effectively controlled cariogenic plaque, thus supporting their use as an adjunctive measure in oral hygiene regimes. These studies collectively underscore the nuanced roles of different oral hygiene aids in managing oral health among orthodontic patients, highlighting the importance of comprehensive care strategies that go beyond the type of toothbrush or mouthwash used.

A primary limitation of the study is the cross-sectional design, preventing definitive conclusions on causality between toothbrush usage, mouthwash regimens, and halitosis outcomes. While we observed robust associations, prospective or interventional studies could better delineate cause-and-effect relationships. Second, the organoleptic test, although widely considered a gold standard, can be influenced by examiner bias and nasal fatigue, underscoring the potential utility of instrumental measures like gas chromatography for future research. Third, although the sample size was reasonable for the primary comparisons, certain subgroup analyses (particularly for specific mouthwash types) contained modest numbers, which might limit statistical power. Fourth, self-reported oral hygiene adherence and potential recall bias regarding mouthwash usage can confound the findings. Finally, our data were drawn from two university-based clinics, which might not fully capture the diversity of orthodontic patients in private practices or different socioeconomic contexts. Expanding such investigations to broader populations would strengthen external validity.

We recognize that external factors—dietary habits, smoking status in older adolescents or young adults, and systemic conditions (e.g., GERD)—can significantly affect halitosis. Although our exclusion criteria incorporated major systemic diseases and recent antibiotic use, we did not formally track smoking or dietary patterns. Future studies should incorporate these variables in a multivariate model to better isolate the impact of brushing technique and mouthwash choice on halitosis.

## 5. Conclusions

From a clinical standpoint, the results of this study suggest three key recommendations for orthodontists to consider. Firstly, promoting the use of sonic toothbrushes along with chlorhexidine-based mouthrinses could significantly reduce halitosis, especially for those susceptible to plaque retention. Secondly, it is crucial to emphasize continued oral hygiene compliance after the removal of orthodontic appliances; residual plaque niches or dry mouth can sustain halitosis, and regular check-ups along with reinforcement of proper brushing and rinsing techniques may help to mitigate persistent malodor. Lastly, while the cross-sectional design of our study identified associations, future research in the form of prospective randomized controlled trials, with detailed tracking of potential confounders such as diet, smoking habits, and systemic diseases, are warranted to validate these findings and further refine best-practice guidelines for orthodontic patients. By implementing these recommendations, clinicians can enhance patient education, optimize plaque control, and improve the psychosocial well-being of patients related to halitosis during and after orthodontic treatment.

## Figures and Tables

**Table 1:** Participant demographics and orthodontic status.

Variable	Traditional (*n* = 57)	Rotative (*n* = 64)	Sonic (*n* = 53)	Total (*n* = 174)	*p*-Value
Age (years), mean ± SD	17.8 ± 1.3	18.2 ± 1.6	18.0 ± 1.4	18.0 ± 1.5	0.377
Female, *n* (%)	34 (59.6)	40 (62.5)	33 (62.3)	107 (61.5)	0.921
Male, *n* (%)	23 (40.4)	24 (37.5)	20 (37.7)	67 (38.5)	-
Duration of Tx (months)	14.7 ± 2.8	15.3 ± 2.7	14.4 ± 2.6	14.8 ± 2.7	0.46
“During” appliances, *n* (%)	34 (59.6)	36 (56.3)	28 (52.8)	98 (56.3)	0.679
“After” appliances, *n* (%)	23 (40.4)	28 (43.8)	25 (47.2)	76 (43.7)	-

**Table 2:** HALT summary scores by toothbrush group.

HALT Domain	Traditional (*n* = 57)	Rotative (*n* = 64)	Sonic (*n* = 53)	*p*-Value (ANOVA)
Emotional Impact (0–25)	14.2 ± 3.2	12.6 ± 3.1	10.9 ± 2.8	0.001
Social/Interactional (0–25)	11.4 ± 2.6	10.3 ± 2.5	9.4 ± 2.3	0.006
Personal/Functional (0–25)	13.1 ± 3.0	11.6 ± 2.9	10.7 ± 2.5	0.004
Physical Concerns (0–25)	4.3 ± 2.2	3.6 ± 1.9	3.3 ± 1.7	0.047
Total HALT (0–100)	42.7 ± 6.2	38.1 ± 6.0	34.8 ± 5.8	<0.001

Post hoc (Tukey) findings: Traditional vs. Rotative: *p* = 0.013 for total HALT; Traditional vs. Sonic: *p* < 0.001 for total HALT; Rotative vs. Sonic: *p* = 0.004 for total HALT.

**Table 3:** SF-36 domain scores by toothbrush group.

SF-36 Domain	Traditional (*n* = 57)	Rotative (*n* = 64)	Sonic (*n* = 53)	*p*-Value (ANOVA)
Physical Functioning	82.3 ± 5.3	83.9 ± 4.4	85.1 ± 5.2	0.046
Role Physical	78.5 ± 7.1	80.3 ± 6.2	81.9 ± 6.4	0.049
Bodily Pain	76.7 ± 6.8	78.0 ± 6.4	79.8 ± 6.2	0.036
General Health	73.8 ± 6.2	76.6 ± 5.7	77.7 ± 6.0	0.031
Vitality	75.3 ± 5.6	77.6 ± 5.1	79.0 ± 5.0	0.01
Social Functioning	80.0 ± 4.5	82.2 ± 4.3	84.3 ± 4.2	0.001
Role Emotional	76.8 ± 6.2	77.7 ± 5.9	79.3 ± 5.6	0.098
Mental Health	79.3 ± 5.7	80.5 ± 5.4	82.1 ± 5.2	0.046

**Table 4:** Subgroup analysis: mouthwash use vs. no mouthwash.

Variable	Mouthwash (*n* = 102)	No Mouthwash (*n* = 72)	*p*-Value
HALT Total (0–100)	35.1 ± 6.0	39.2 ± 6.3	<0.001
SF-36 Social	83.7 ± 4.3	80.2 ± 4.8	0.002
Organoleptic Score (0–5)	1.7 ± 0.4	2.1 ± 0.5	<0.001

**Table 5:** Subgroup analysis by mouthwash type.

Mouthwash Type	*n*	HALT Total (Mean ± SD)	Organoleptic Score (Mean ± SD)
Chlorhexidine-based	40	34.3 ± 5.7	1.5 ± 0.4
Essential Oil-based	31	35.7 ± 5.9	1.8 ± 0.5
Fluoride-based	31	36.4 ± 5.6	1.9 ± 0.4
Overall *p*-value	-	0.039	0.033

Post hoc (Tukey) findings (HALT total): Chlorhexidine vs. Fluoride-based: *p* = 0.046; Chlorhexidine vs. Essential Oil-based: *p* = 0.081; Essential Oil-based vs. Fluoride-based: *p* = 0.515.

**Table 6:** Comparison of “during” vs. “after” orthodontic appliance removal.

Timing	*n*	HALT Total (Mean ± SD)	Organoleptic (Mean ± SD)	SF-36 Social (Mean ± SD)
During (Active Tx)	98	39.2 ± 6.4	2.2 ± 0.5	81.2 ± 4.4
After (Removed < 6 months)	76	35.5 ± 5.9	1.7 ± 0.4	83.7 ± 4.3
*p*-values		0.002	<0.001	0.015

**Table 7:** Correlation analysis.

	HALT Total	SF-36 Social	Organoleptic Score	Age	Tx Duration
HALT Total	1	−0.49 *	+0.54 *	0.09	0.16
SF-36 Social	−0.49 *	1	−0.44 *	−0.10	−0.12
Organoleptic Score	+0.54 *	−0.44 *	1	−0.07	0.13
Age	0.09	−0.10	−0.07	1	+0.30 *
Tx Duration	0.16	−0.12	0.13	+0.30 *	1

Spearman’s correlation coefficients shown; all significant correlations (*p* < 0.001) are marked with an asterisk (*). Non-asterisked correlations have *p* > 0.05.

## Data Availability

Data availability are subject to hospital approval.
